# High Co-Expression of *GPAT4* and *SLC7A11* as a Predictor of Platinum Resistance and Poor Prognosis in Patients with Epithelial Ovarian Cancer

**DOI:** 10.3390/biomedicines13071664

**Published:** 2025-07-08

**Authors:** Ping Yu, Chunliang Shang, Zhongyu Liu, Yuan Li, Tianhui He, Yuan Xue, Jian Lin, Yuan Li, Yu Wu, Tong Liu, Hongyan Guo

**Affiliations:** 1 Department of Obstetrics and Gynecology, Peking University Third Hospital, Beijing 100191, China; yuping1996@163.com (P.Y.); shangchl@bjmu.edu.cn (C.S.); liuzhongyudayu@163.com (Z.L.); svenja9528@163.com (T.H.); yuanli@bjmu.edu.cn (Y.L.); pheonix1997@163.com (Y.W.); 2 Center of Basic Medical Research, Institute of Medical Innovation and Research, Cancer Center of Peking University Third Hospital, Peking University Third Hospital, Beijing 100191, China; 3 Reproductive Medicine Center, The University of Hong Kong-Shenzhen Hospital, Shenzhen 518000, China; yuanleedr@163.com; 4 Department of Pharmacy, Peking University Third Hospital, Beijing 100191, China; 2211110545@stu.pku.edu.cn (Y.X.); linjian@pku.edu.cn (J.L.)

**Keywords:** ferroptosis, *GPAT4*, *SLC7A11*, platinum resistance, ovarian cancer

## Abstract

**Background/Objectives:** This study aimed to determine whether the expression levels of *GPAT4* and *SLC7A11* are associated with survival outcomes and platinum resistance in epithelial ovarian cancer (EOC) patients. **Methods:** We analyzed the medical records of EOC patients. EOC samples obtained during surgery were stained for *GPAT4* and *SLC7A11*. Cox regression and Kaplan—Meier analyses were performed to assess the impact of *GPAT4* and *SLC7A11* expression on overall survival (OS). **Results:** We found that *GPAT4* and *SLC7A11* expression levels were greater in platinum-resistant ovarian cancer tissues than in platinum-sensitive ovarian cancer tissues. High expression of both *GPAT4* and *SLC7A11* was associated with an increased risk of platinum resistance compared with low expression of both factors. High expression of both SLC7A11 and GPAT4 was independently correlated with poor OS, highlighting the significance of this integrated metric as a prognostic factor in ovarian cancer. The *GPAT* inhibitor (GPAT-IN-1) and an *SLC7A11* inhibitor (erastin) attenuated platinum resistance in ovarian cancer cells, and their combined application increased cytotoxicity. Furthermore, the combination of GPAT-IN-1, erastin, and cisplatin significantly improved the chemotherapeutic effects on platinum-resistant ovarian cancer cells. **Conclusions:** High expression of both *SLC7A11* and *GPAT4* is related to platinum resistance in EOC patients. The high expression of both *SLC7A11* and *GPAT4* serves as an important independent prognostic factor and indicates potential therapeutic targets for patients with platinum-resistant EOC.

## 1. Introduction

Ovarian cancer is a malignant tumor with the highest recurrence and mortality rates among cancers of the female reproductive system [1,2]. Notably, chemotherapy resistance is a leading cause of death among ovarian cancer patients. Although maintenance drug therapy with poly (ADP-ribose) polymerase inhibitors [3] can target homologous recombination defects [4,5,6], antiangiogenic agents such as bevacizumab and PD-1/PD-L1 inhibitors are widely used for treating epithelial ovarian cancer (EOC) [7]. Nonetheless, platinum-based chemotherapy remains the cornerstone treatment for EOC [8]. However, the incidence of platinum resistance is high and has not yet been effectively addressed in the clinic. Thus, there is an urgent need to identify key targets for ovarian cancer resistance and improve patient-specific treatment strategies to address treatment resistance and improve the prognosis of ovarian cancer.

Ferroptosis, a recently defined cell death, is a key factor in redox balance [9], lipid metabolism imbalance, and lipid peroxide accumulation in cells. In recent years, numerous studies have shown that ferroptosis is involved in the resistance of malignant tumors to chemotherapy and is associated with tumor prognosis [10,11,12].

Platinum can trigger ferroptosis in tumor cells. The mechanisms may include the following: the consumption of intracellular glutathione; the inhibition of intracellular glutathione peroxidase activity, leading to ferroptosis; an increase in the availability of free iron, which is transported to lysosomes to activate ferroptosis, after ferritin autophagy; and the accumulation of many lipid peroxides, leading to the acceleration of the Fenton reaction, which can trigger ferroptosis [13].

Platinum resistance in ovarian cancer patients is associated with a poor prognosis. In other words, ovarian cancer cells are resistant to ferroptosis, which may be due to the occurrence of protective ferroptosis mechanisms in the cells, such as the activation of antioxidant signaling pathways to escape ROS stress and reduce the formation of lipid peroxides [14]. Alternatively, an increase in ferroptosis inhibitory factor 1 (*FSP1*) N-nutmeg acylation can inhibit its degradation and cell membrane translocation and increase the resistance of tumor cells to iron [15].

Ferroptosis sensitivity is closely related to changes in lipid metabolism [16]. Different fats play a role in epicuticular ferroptosis: various polyunsaturated phospholipid fatty acids, such as arachidonic acid, interact with ROS to induce ferroptosis [17,18]. In contrast, overexpression of stearoyl-CoA desaturase 1 (*SCD1*) promotes the production of monounsaturated fatty acid (MUFA) phospholipids [19,20], which can suppress ROS-induced stress.

Glycerol-3-phosphate acyltransferase 4 (*GPAT4*) is an endoplasmic reticulum membrane protein and the initial rate-limiting enzyme that catalyzes the conversion of glycerol-3-phosphate to saturated lysophosphatidic acids (LPAs) [21,22]. LPAs are bioactive phospholipids that are present at elevated concentrations in the tumor microenvironment and ovarian cancer ascites [23,24,25], stimulate cancer cell growth and tissue invasion, and are associated with drug resistance in ovarian cancer [26]. Lysophosphatidic acid receptor 3 (LPA3) activation can inhibit ferroptosis by inhibiting lipid oxidation and iron accumulation [27]. High expression levels of *GPAT4* may be linked to ferroptosis and platinum resistance in ovarian cancer. *SLC7A11* is a cystine transporter that controls drug influx into a key transporter and is a member of the solute carrier superfamily [28,29]. SLC7A11 interacts with SLC3A2 in the heterodimeric amino acid transport system x (c), which mediates cystine–glutamate exchange and maintains intracellular glutathione levels, resulting in cisplatin resistance in ovarian cancer cells [30,31]. *SLC7A11*, as an inhibitor of ferroptosis, may be a new therapeutic target for platinum-resistant ovarian cancer patients. Recent studies have identified *SLC7A11*, a key component of amino acid metabolism, as a potential biomarker for platinum resistance in ovarian cancer [32]. While elevated *SLC7A11* expression is associated with a poor prognosis in ovarian cancer patients, its diagnostic performance remains suboptimal. Notably, ferroptosis is regulated by amino acid metabolism and is also tightly linked to lipid metabolism. In this study, we make a report that is the first of its kind. We present evidence that *GPAT4*, a gene linked to both lipid metabolism and ferroptosis, may also play a role in ovarian cancer’s resistance to platinum-based treatments.

This study aimed to investigate the diagnostic value of high *GPAT4* expression combined with high *SLC7A11* expression in patients with platinum-resistant EOC and provide an experimental basis for further studies of the biopharmaceutical antitumor applications of *GPAT4* inhibitors and *SLC7A11* inhibitors.

## 2. Materials and Methods

### 2.1. Study Design and Patient Characteristics

This research included platinum-resistant OC patients admitted to Peking University Third Hospital between January 2016 and December 2021. This study was approved by the Ethics Committee of the Peking University Third Hospital (M2019291).

Patients were eligible if they met the following criteria:

- For ovarian cancer staging surgery or tumor cell reduction in our hospital, all samples were collected from the initial cytoreductive surgery in the ovary.
- Patients had a histological diagnosis of EOC, and pathological tissue sections were obtained.
- Imaging was performed before surgery, and patients had intraoperatively measurable lesions.
- Postoperative pathology revealed epithelial ovarian cancer, and platinum-based chemotherapy was performed after surgery.
- There was no history of other malignant tumors.
- Patients had complete clinical data.
- Complete follow-up information was available for all included patients.

The exclusion requirements are as follows:

- The quality of the pathological tissue sections was not good.
- Postoperative routine pathology revealed other types of tumors rather than ovarian epithelial carcinoma.
- Regular chemotherapy was not performed after surgery.
- The patient had other basic diseases that affect the survival of patients.
- The patient received neoadjuvant chemotherapy.
- The patient was pregnant or breastfeeding.

The platinum-free interval (PFI) was defined as the duration between the implementation of the final platinum-based chemotherapy and the beginning of illness. The EOC patients were divided into a platinum-resistant group (<six months PFI) and a platinum-sensitive group (≥six months PFI). Follow-up was implemented via telephone communication and outpatient review.

The platinum-resistant group was further filtered to select patients who did not respond to first-line platinum-based chemotherapy (that is, those with primary platinum resistance); the control group consisted of platinum-sensitive patients matched at a 1:2 ratio on the basis of FIGO stage, age, histological subtype, and tumor grade.

The following clinical data were collected for all the subjects: ascites volume, age, preoperative serum CA125 level, International Federation of Obstetrics and Gynecology (FIGO) stage, tumor grade, date of first surgery, type of surgery, and time of paclitaxel combined with cisplatin or carboplatin (TP) regimen completion. On the basis of the GOG-172 conditions, surgery was categorized as best cell reduction (residual lesion < 1.0 cm) or suboptimal cell reduction (residual lesion > 1.0 cm). In accordance with the 2014 FIGO guidelines, tumors removed during surgery were categorized as stage I-IV. The methods adopted to evaluate overall survival (OS) and progression-free survival (PFS) were the same as those used in past studies.

### 2.2. 5hmC Library Construction and High-Throughput Sequencing

Ovarian cancer tissue samples were obtained from Peking University Third Hospital (ethical approval number M2019291). Genomic DNA (gDNA) was extracted from these tissues using Quick-DNATM miniprep Plus Kit (ZYMO, Irvine, CA, USA; Cat# D4069) and quantified by Qubit3.0 (Thermo, Waltham, MA, USA; Cat# Q33216). gDNA was treated with non-restriction endonucleases followed by nucleic acid electrophoresis to assess fragment size (1000~bp), after which fragments were sent to Professor Jian Lin’s laboratory for establishment of the 5hmC library and subsequent high-throughput sequencing [33].

### 2.3. Immunohistochemistry

Immunohistochemical analysis of formalin-fixed, paraffin-embedded (FFPE) tissue samples was performed for eligible EOC patients. The FFPE slices (5 μm thick) were dewaxed in xylene, rehydrated in a graded ethanol series, and quenched with endogenous peroxide. The sections were subsequently incubated with anti-*SLC7A11* (1:100 NCM universal antibody diluent ab37185; Abcam, Cambridge, UK) and anti-*GPAT4* (1:100, ab76707 in NCM Universal Antibody diluent; Abcam, Cambridge, UK) antibodies. Two consecutive sections from the paraffin-embedded samples were stained with anti-*GPAT4* and anti-*SLC7A11* antibodies. The sections were reverse-stained with hematoxylin, dehydrated, and fixed. The negative controls consisted of tumor samples that contained 75% tumor tissue and were incubated without primary antibodies as previously described. *GPAT4* and *SLC7A11* were evaluated semi-quantitatively as the percentage of positive cells for each protein among all ovarian cancer cells for all sections. The scores for the percentage of positively stained cells ranged from 1 to 4: 1 means zero to twenty-five percent, 2 means twenty-six to fifty percent, 3 means fifty-one to seventy-five percent, and 4 represents seventy-six to one hundred percent. The staining intensity scores ranged from 0 to 3: strong = 3, medium = 2, negative = 0, and weak = 1. The product of the percentage and intensity scores was used as the overall score and ranged from zero to twelve. High expression was defined as a score between five and twelve points, and low expression was defined as a score between zero and four points. All pathological diagnoses and staining outcomes were documented by two specialist pathologists who were blinded to the clinical data of the patients.

### 2.4. Cell Culture

SKOV3 DDP and A2780 DDP cells were obtained from Neovander Co., Ltd. (Beijing, China) in October 2020 and June 2021. A2780 DDP and SKOV3 DDP cells were cultured in ATCC-formulated Dulbecco’s modified Eagle’s medium (Gibco, Thermo Fisher Scientific, Waltham, MA, USA), which contains ten percent fetal bovine serum (FBS, Gibco, Thermo Fisher Scientific, Waltham, MA, USA), one percent penicillin-streptomycin, and 0.5 μ/mL cisplatin (Sigma-Aldrich, Merck KGaA, Darmstadt, Germany) at 37 °C and 5% CO_2_. The mixed cells are passed in a ratio of 1:4 or 1:5.

### 2.5. The Cell Viability Was Assessed by IncuCyte Live-Cell Assays

SKOV3 DDP and A2780 DDP cells’ viability was assessed via IncuCyte live-cell assays (Sartorius Incucyte S3). SKOV3 DDP cells were plated in 96-well plates at a density of 3 × 10^3^ cells/well after counting with a hemocytometer. A2780 DDP cells were plated in 96-well plates at a density of 5 × 10^3^ cells/well. Cell proliferation was assessed via IncuCyte live-cell assays after treatment with cisplatin, GPAT-IN-1 (*GPAT* isoform inhibitor), and erastin (*SLC7A11* inhibitor) for 72 h. IncuCyte live-cell assays took pictures every 3 h through continuous collection and counted the number of cells for quantitative analysis.

### 2.6. Statistical Analysis

SPSS (version 23.0) (IBM Corp.) was used for analysis. The Kolmogorov–Smirnov test is adopted to determine the normality of continuous data. For normally distributed data, the Student’s *t*-test was used to compare two independent groups, while the Mann-Whitney U test was applied to non-normally distributed data. The chi-square test was used to compare unpaired categorical data between groups. Fisher’s exact test was applied to analyze contingency tables for categories with fewer than five subjects. To evaluate potential multicollinearity among the independent variables used in logistic and Cox regression analyses, we calculated the Variance Inflation Factor (VIF) for each predictor. The variables assessed included age, FIGO stage, *GPAT4* expression, *SLC7A11* expression, and *GPAT4* and *SLC7A11* co-expression. All variables demonstrated VIF values well below the commonly accepted threshold of 5 (range: 1.05–1.63), indicating no significant multicollinearity concerns. Logistic regression models were used to classify the dominance of risk factors. Dependent Variable: Platinum response status; independent Variables: GPAT4 expression, SLC7A11 expression, FIGO stage (I–II vs. III–IV), and surgical outcome (R0/R1 vs. R2); categorization: Age (<50 vs. ≥50 years), GPAT4 and SLC7A11 (high vs. low expression based on IHC scores).

Correlation analysis involved scatterplot analysis and Spearman’s correlation coefficient (two-tailed). OS curves were assessed via the Kaplan–Meier method, and survival differences were estimated via the log-rank test. Univariate and multivariate Cox analyses were then implemented, incorporating total potential predictors. Dependent Variables: Overall survival (OS) and progression-free survival (PFS); independent Variables: Age, baseline CA125 level, CA125 normalization after three cycles of chemotherapy, FIGO stage, ascitic fluid volume, *GPAT4* expression, *SLC7A11* expression, and platinum resistance.

Receiver Operating Characteristic (ROC) curves were employed to determine the potential predictive value of factors assessed in the comparative analysis above. ROC analysis was performed to assess the diagnostic performance of *GPAT4* expression, *SLC7A11* expression, and their co-expression status in predicting platinum resistance in ovarian cancer. The outcome variable was platinum response status (platinum resistant vs. platinum sensitive), while the predictor variables included IHC scores (continuous) and binary groupings (high vs. low expression) of *GPAT4* and *SLC7A11*. If the 95% confidence interval (CI) of the scope under the curve (AUC) is low (>0.5), the factor was considered significant. A *p* value less than 0.05 (two-tailed) indicated a statistically significant difference.

## 3. Results

### 3.1. Screening of GPAT4

**Figure 1:**
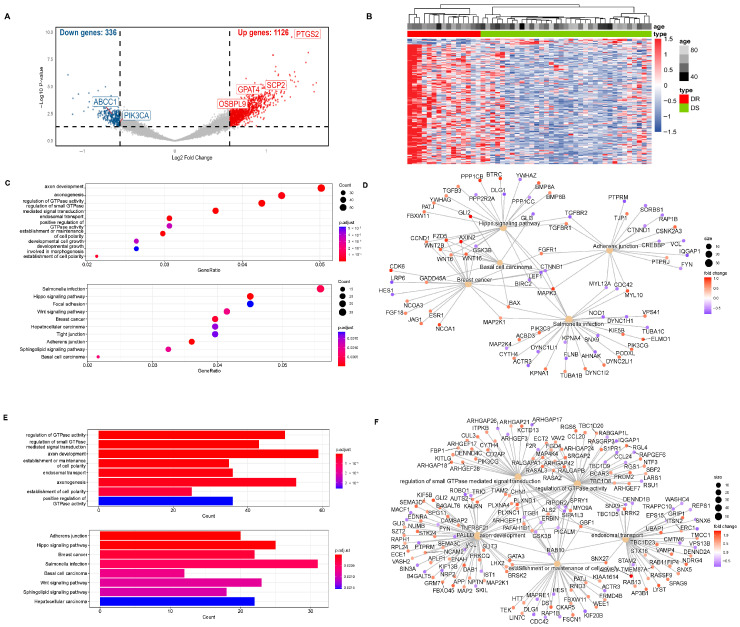
5hmC hydroxymethylation sequencing was performed on platinum-resistant and platinum-sensitive ovarian cancer patients. (**A**) 5hmC hydroxymethylation sequencing was performed on 50 patient ovarian cancer tissues, including 35 platinum-sensitive ovarian cancers and 15 platinum-resistant ovarian cancers. All 1106 differentially hydroxymethylated expressed genes (DhMGs) were found in the volcano map. (**B**) A heatmap was constructed to compare the top 200 DhMGs between platinum-sensitive and platinum-resistant ovarian cancers (DR: platinum-resistant ovarian cancer, DS: platinum-sensitive ovarian cancer). (**C**) Total number of different genes between platinum-sensitive and platinum-resistant ovarian cancer from KEGG Enrichment of bubble chart. (**D**) Totally different genes between platinum-sensitive and platinum-resistant ovarian cancer in KEGG functional network diagram. (**E**) Total number of different genes between platinum-sensitive and platinum-resistant ovarian cancer of GO Enrichment bar graph. (**F**) Total number of different genes between platinum-sensitive and platinum-resistant ovarian cancer in GO functional network diagram.

**Figure 2:**
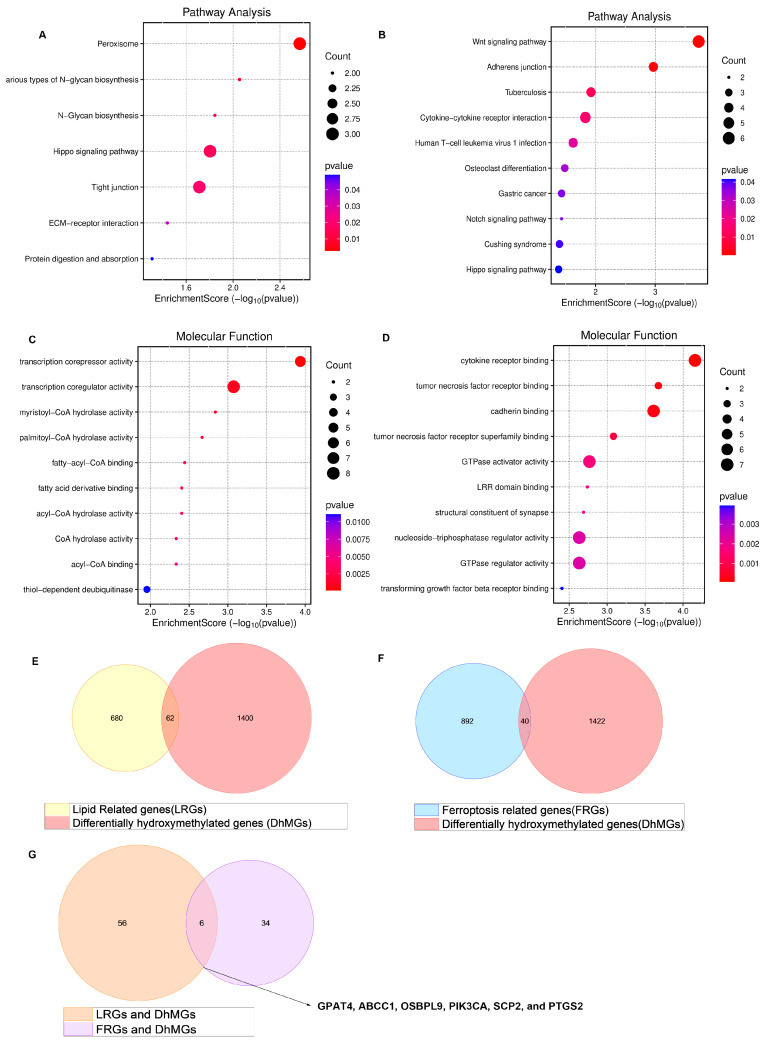
Enrichment of all different genes discovered by 5hmC hydroxymethylation sequencing, including platinum-resistant genes related to ferroptosis and lipid metabolism. (**A**) 5hmC hydroxymethylation sequencing discovered the different up top 100 DhMGs related to platinum resistance in ovarian cancer in pathway of bubble chart. (**B**) 5hmC hydroxymethylation sequencing discovered the different down top 100 DhMGs related to platinum resistance in ovarian cancer in pathway of bubble chart. (**C**) 5hmC hydroxymethylation sequencing discovered the different up top 100 DhMGs related to platinum resistance in ovarian cancer in the molecular function of bubble chart. (**D**) 5hmC hydroxymethylation sequencing discovered the different down top 100 DhMGs related to platinum resistance in ovarian cancer in the molecular functional bubble chart. (**E**) 5hmC hydroxymethylation sequencing discovered the different down genes related to platinum resistance in ovarian cancer of KEGG enrichment of bubble chart. (**E**) Venn diagram displaying all DhMGs discovered by 5hmC hydroxymethylation sequencing, 742 genes related to lipid metabolism GSEA/MSigDB. (**F**) Venn diagram displaying all DhMGs discovered by 5hmC hydroxymethylation sequencing, the FerrDb gene bank (FerrDB V2, http://www.zhounan.org/ferrdb/current/ (accessed on 1 March 2023)) related to ferroptosis. (**G**) Venn diagram displaying 6 genes that overlap by DhMGs, LRGs, and FRGs, including *GPAT4*, *ABCC1*, *OSBPL9*, *PIK3CA*, *SCP2*, and *PTGS2*.

Our research group identified 1462 differentially hydroxymethylated genes (DhMGs) from 35 platinum-sensitive ovarian cancer samples and 15 platinum-resistant ovarian cancer tissue samples by 5hmC hydroxymethylation sequencing (|log2 Fold change| > 0.6, *p* < 0.05, Figure 1A). A heatmap was constructed to compare the top 200 DhMGs between platinum-sensitive and platinum-resistant ovarian cancer (Figure 1B). Using the R package 4.2.2 “limma,” we detected platinum-resistance-related DhMGs and performed functional enrichment analysis to investigate the probable biological behavior of platinum-resistant ovarian cancer (Figure 1C–F). Next, we detected platinum-resistant-related up-DhMGs and down-DhMGs and performed functional enrichment analysis to investigate the probable biological behavior (Figure 2A–D). Additionally, through overlap with genes related to ferroptosis and lipid metabolism, we identified six relevant genes obtained by crossing, including *GPAT4*, *ABCC1*, *OSBPL9*, *PIK3CA*, *SCP2,* and *PTGS2* (Figure 2G). After analyzing the prognosis and survival of ovarian cancer patients with protein expression of these six genes in the TCGA, we found that only high expression of *GPAT4* was associated with poor prognosis in ovarian cancer patients. Next, we focused on the target of the key enzyme of lipid metabolism, *GPAT4*, in promoting tumor growth through ferroptosis in platinum-resistant ovarian cancer cells. Notably, although one cluster in the 5hmC-based heatmap exhibited clear separation of platinum-resistant samples, other clusters contained a mixture of resistant and sensitive cases (Figure 1B). This heterogeneity may reflect early or partial epigenetic reprogramming within platinum-sensitive tumors. Given that 5hmC is a dynamic epigenetic mark associated with gene regulation, certain sensitive tumors may share hydroxymethylation patterns with resistant tumors prior to overt phenotypic resistance. In addition, variations in tumor purity, stromal infiltration, and cell composition could also contribute to the observed clustering pattern. Technical variables, such as DNA quality and bisulfite conversion efficiency, may further introduce noise, emphasizing the importance of integrating 5hmC profiles with functional validation.

### 3.2. Association of GPAT4 and SLC7A11 Expression with Clinical Features and Platinum Resistance

To further validate the 5hmC-seq findings, a total of 134 ovarian cancer patients were initially recruited. Nineteen patients whose tumor tissue percentage was <70% and 13 patients whose staining results were unsatisfactory were excluded; the final cohort consisted of 102 patients (Tables S1–S3). The average age of enrolled patients was 54 years (scope: 24–77 years). Two patients (1.96%, 2/102) had highly differentiated tumors, three patients (2.94%, 3/102) had moderately differentiated tumors, and the remaining 97 cases (95.10%, 97/102) had poorly differentiated tumors. Most cases (88.24%, 96/102) were classified as high-grade serous ovarian cancer. Most cases (81.11%, 83/102) were classified as FIGO stage III or IV, whereas the remaining 12 (11.76%, 12/102) were categorized as FIGO stage I or II.

*GPAT4* was selected for further analysis based on its significant differential hydroxymethylation in platinum-resistant ovarian cancer and its reported involvement in ferroptosis and lipid metabolism. Another ferroptosis-related gene, *SLC7A11*, which has been implicated in platinum resistance, was also included for validation. The potential diagnostic and therapeutic roles of *GPAT4* and *SLC7A11* in ovarian cancer are explored further in subsequent sections. High expression of *GPAT4* was significantly associated with suboptimal primary debulking surgery (*p* < 0.05). Notably, elevated expression of both GPAT4 and SLC7A11 was strongly correlated with platinum resistance (*p* < 0.05).

### 3.3. Role of SLC7A11 and GPAT4 Expression in the Prediction of Drug Resistance in Ovarian Cancer

**Figure 3:**
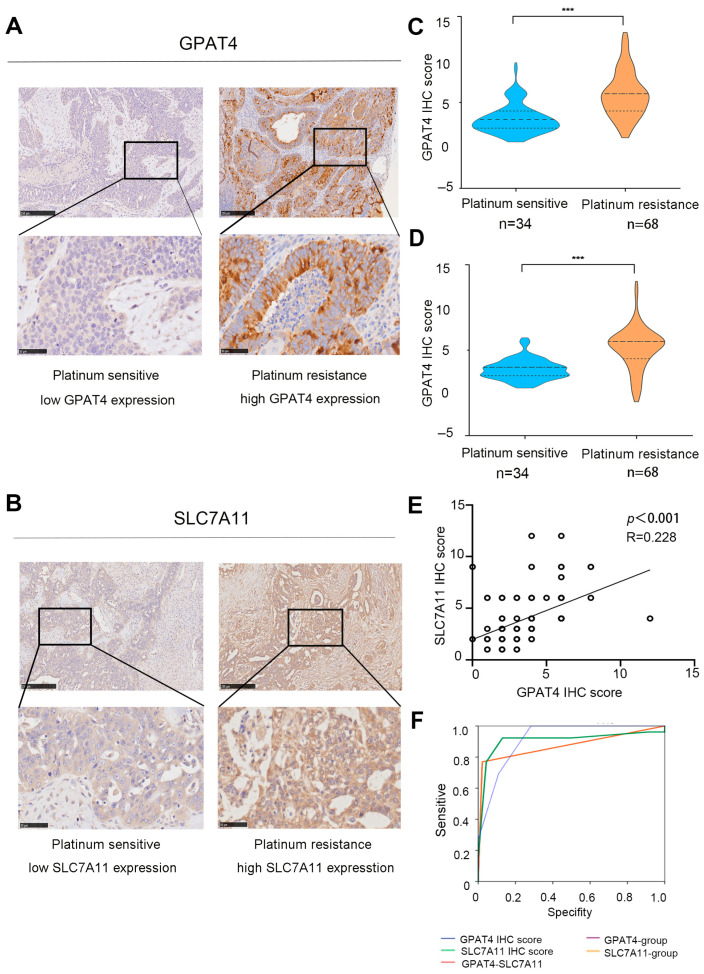
Expression of *SLC7A11* and *GPAT4* in ovarian cancer. (**A**) Representative micrographs of GPAT4 staining in platinum-resistant and platinum-sensitive tissues from ovarian cancer patients scale bars (50 and 250 microns) are displayed at the bottom left. (**B**) Representative micrographs of SLC7A11 staining from platinum-sensitive tissues and platinum-resistant tissues from ovarian cancer patients. Scale bars (fifty and two hundred and fifty micrometers) are shown on the bottom left. (**C**) *GPAT4* IHC score in platinum-sensitive and platinum-resistant ovarian cancer tissues. (**D**) *SLC7A11* IHC score in platinum-resistant and platinum-sensitive ovarian cancer tissues. (**E**) Correlations between *GPAT4* immunohistochemical scores and *SLC7A11* immunohistochemical scores. (**F**) Role of *SLC7A11* and *GPAT4* expression in the diagnosis of ovarian cancer. *** *p* < 0.001 compared.

**Figure 4:**
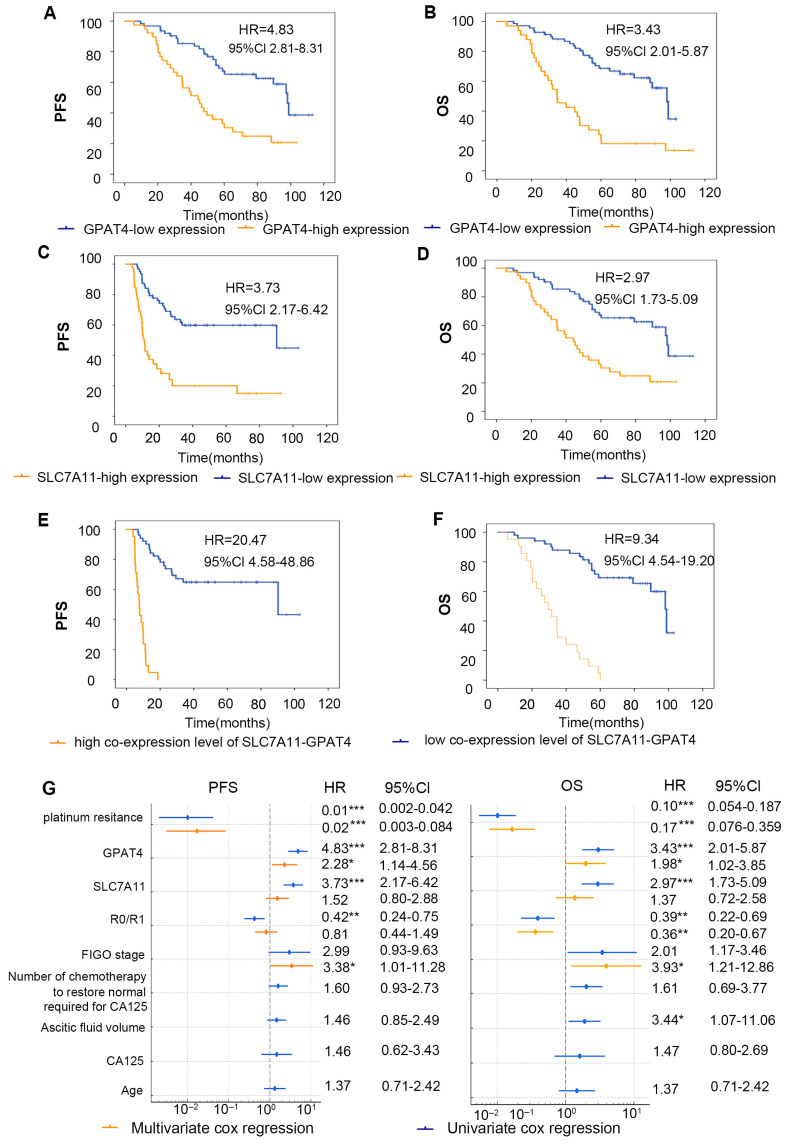
Correlation analysis of *GPAT4* and *SLC7A11* expression and prognosis of patients. (**A**) Kaplan–Meier survival curve of PFS in ovarian cancer patients with diverse GPAT4 expression levels. (**B**) Kaplan–Meier survival curves for PFS in ovarian cancer patients with various *SLC7A11* expression levels. (**C**) Kaplan–Meier survival curves for OS in ovarian cancer patients with various *GPAT4* expression levels. (**D**) Kaplan–Meier survival curves for OS in ovarian cancer patients with various *SLC7A11* expression levels. (**E**) Kaplan–Meier survival curves for OS in ovarian cancer patients with various *co-SLC7A11-GPAT4* expression levels. (**F**) Kaplan–Meier survival curves for OS in ovarian cancer patients with various *co-SLC7A11-GPAT4* expression levels. (**G**) Forest plots of multivariate and univariate Cox regression analysis. * *p* < 0.05; ** *p* < 0.01; *** *p* < 0.001.

According to the immunohistochemical staining results, *GPAT4* expression in platinum-resistant ovarian cancer (*n* = 34) was significantly increased compared to that in platinum-sensitive tissues (*n* = 56, *p* < 0.001) (Figure 3A,C). Specifically, *GPAT4* had a median IHC mark of 2 in platinum-sensitive ovarian cancer and a median IHC mark of 6 in platinum-resistant ovarian cancer. Immunohistochemical analysis of *SLC7A11* in ovarian cancer tissue revealed a median IHC score of 6 for *SLC7A11* in platinum-resistant ovarian cancer and a median IHC score of 4 in platinum-sensitive ovarian cancer (Figure 3B,D, *p* < 0.001). As an individual indicator, *GPAT4* exhibited a strong predictive capability for drug resistance in ovarian cancer (*GPAT4* IHC score AUC 0.917, GPAT4 expression group AUC 0.874, Table S4, Figure 4F). In this study, *GPAT4* was significantly superior to SLC7A11 as a stand-alone predictor of platinum-resistant ovarian cancer drug resistance (SLC7A11 IHC score AUC 0.908, SLC7A11 expression grouping AUC 0.874, Table S4).

Furthermore, a Spearman correlation analysis further revealed a correlation between GPAT4 immunohistochemical scores and *SLC7A11* immunohistochemical scores (Figure 3E; *r* = 0.228, *p* < 0.001). While the combined assessment of *GPAT4* and *SLC7A11* showed improved predictive ability compared with *SLC7A11* alone, it was not as effective as using *GPAT4* alone for predicting drug resistance in ovarian cancer. These findings indicate that *GPAT4* is the most reliable predictor of drug resistance in epithelial ovarian cancer.

### 3.4. Effects of GPAT4 and SLC7A11 Expression on the Prognosis of Ovarian Cancer

To evaluate the impact of the combined IHC score grouping of *SLC7A11* and *GPAT4* on the prognosis of ovarian cancer patients, we categorized the tissues that tested positive for both *SLC7A11* and *GPAT4* as the copositive group (*n* = 21). We also grouped patients with negative *GPAT4* with SLC7A11 co-expression negative groups (n = 51) and analyzed the two groups (Figure 4E,F). In general, 32 out of 34 patients in the platinum-resistant group died (94.12%, 32/34), whereas 23 of 68 patients in the platinum-sensitive group died (33.82%, 23/68) (*p* < 0.001). A total of 55 deaths occurred; one patient died of a stroke 27 months after surgery without any return of ovarian cancer. We calculated the OS for 102 patients and found that the high expression of *GPAT4* group median survival time was 34.7 months and low expression of *GPAT4* group median survival time was 98.1 months. High expression of *SLC7A11* group median survival time was 44.3 months and low expression of *SLC7A11* group median survival time was 98.0 months. The high expression of *GPAT4* or *SLC7A11* has been demonstrated to correlate with a poor prognosis in ovarian cancer patients from the TCGA-OC cohorts (Figure S1A,B). The results demonstrated a similar outcome (Figure 4A,B). Furthermore, in comparison to the individual parameters (GPAT4 or SLC7A11 expression), a high co-expression level of GPAT4-SLC7A11 was identified as a more effective predictor of poor prognosis in ovarian cancer (Figure 4E,F).

### 3.5. Effect of GPAT4 and SLC7A11 Inhibitors on Ovarian Cancer Cells

**Figure 5:**
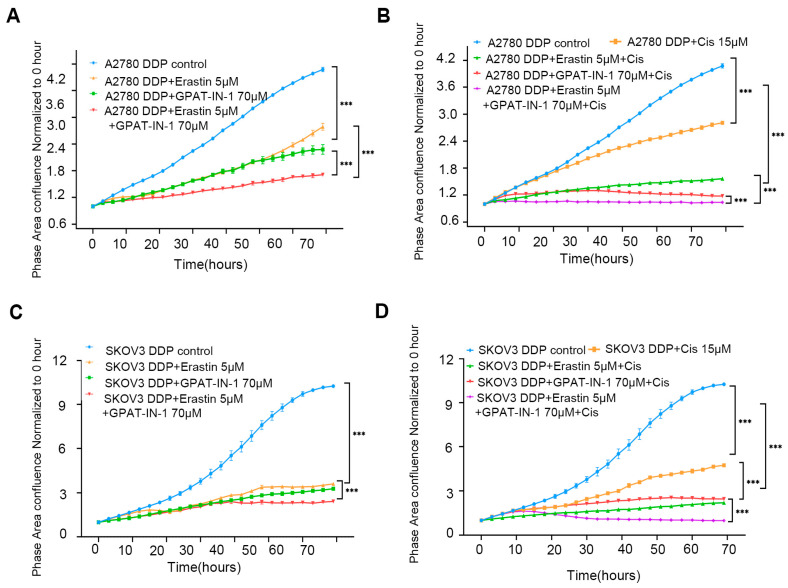
GPAT-IN-1, erastin, and cisplatin on SKOV3 DDP and A2780 DDP cells. (**A**) Validation of the effects of GPAT-IN-1 (a GPAT inhibitor), erastin (SLC7A11 inhibitor), and GPAT-IN-1 combined with erastin on A2780 DDP. A2780 DDP cells were inoculated in 96-well plates at a density of 5 × 10^3^ cells/well, and treated with GPAT-IN-1 or erastin for 72 h. (**B**) Validating the effects of cisplatin, cisplatin combined with GPAT-IN-1, cisplatin combined with erastin, and cisplatin combined with GPAT-IN-1 and erastin on A2780 DDP. (**C**) Validation of the effects of GPAT-IN-1 (GPAT inhibitor), erastin (*SLC7A11* inhibitor), and GPAT-IN-1 combined with erastin on SKOV3 DDP. SKOV3 DDP cells were inoculated in 96-well plates with a density of 3 × 10^3^ cells/well, and treated with GPAT-IN-1 or erastin for 72 h. (**D**) Validating the effects of cisplatin, cisplatin combined with GPAT-IN-1, cisplatin combined with erastin, and cisplatin combined with GPAT-IN-1 and erastin on SKOV3 DDP. Images were taken every 3 h through continuous collection and the number of cells was counted for quantitative analysis. The data are presented as the average number ± SD values (*n* = 3); *** *p* < 0.001 compared with the control group.

The in vitro results revealed that GPAT-IN-125 (a glycerol-3-phosphate acyltransferase isoform inhibitor) and erastin (an *SLC7A11* inhibitor) significantly decreased the viability of ovarian cancer cells and inhibited their proliferation. Compared with either drug alone, the combination of 70 μM GPAT-IN-1 and 5 μM erastin had greater inhibitory effects on SKOV3 DDP cells (Figure 5A) and A2780 DDP cells (Figure 5B). Furthermore, the coadministration of GPAT-IN-1 and erastin markedly increased the cytotoxicity of cisplatin against SKOV3 DDP cells and A2780 DDP (Figure 5C,D).

## 4. Discussion

This study is the first report not only of the predictive value of the ferroptosis-related gene *GPAT4* but also of the value of high expression of both *GPAT4* and *SLC7A11* in predicting platinum resistance in EOC. The combination of GPAT-IN-1 (glycerol-3-phosphate acyltransferase isoforms inhibitor) and erastin (a ferroptosis inducer and an *SLC7A11* inhibitor) significantly enhanced the killing effect of cisplatin on SKOV3 DDP and A2780 DDP cells, which sheds new light on clinical strategies for overcoming platinum-resistant ovarian cancer. The study revealed that *GPAT4/SLC7A11* is related to drug resistance and a poor prognosis in ovarian cancer. The combination of GPAT-IN-1 and erastin was found to increase the cytotoxicity of cisplatin in platinum-resistant ovarian cancer.

Previous work has shown that ferroptosis resistance mechanisms play a significant role in platinum resistance in ovarian cancer [34,35,36,37], and *SLC7A11*, a ferroptosis protective protein, can predict platinum resistance in ovarian cancer patients to some extent. However, as a sole predictor, *SLC7A11* contributes minimally to platinum resistance in ovarian cancer. *GPAT4* plays a crucial role as a key enzyme in fatty acid synthesis, which is an essential process in lipid metabolism [38]. It facilitates the transfer of fatty acids from coenzyme A to glycerol-3-phosphate, which generates triacylglycerol. *GPAT4* inhibitors have been explored as potential targeted therapies that can regulate fatty acid synthesis [39]. However, *GPAT4* inhibitors have rarely been applied in the field of platinum resistance prediction in ovarian cancer. One such inhibitor, GPAT-IN-1, is a glycerol-3-phosphate acyltransferase isoform inhibitor that was found to be cytotoxic to platinum-resistant ovarian cancer. Additionally, the combination of GPAT-IN-1 and erastin with cisplatin was found to induce a more significant chemotherapeutic effect on platinum-resistant ovarian cancer cells than the combination of GPAT-IN-1 or erastin with cisplatin. This study suggested that further screening of patients with drug-resistant ovarian cancer and those with platinum-sensitive ovarian cancer after sequencing may reveal an association between *GPAT4* expression and platinum resistance in ovarian cancer.

These findings further support previous studies linking *SLC7A11* to the prognosis of ovarian cancer [32,40]. However, clinical data and survival outcomes were collected retrospectively, which may introduce bias. The FIGO stage was not found to be associated with *GPAT4* expression, which may be due to the limited sample size in this study. Our follow-up studies will continue to provide insights into the mechanisms by which *GPAT4* regulates platinum resistance in ovarian cancer; we will increase the number of assessed patients with EOC and work to establish a link between *SLC7A11* and the regulation of *GPAT4*. Despite the promising results, several limitations of this study must be considered. First, this was a retrospective, single-center study, which may lead to selection bias during patient recruitment and tissue sampling. Second, the overall sample size—particularly in the platinum-sensitive group—was relatively small, potentially limiting the statistical power and external generalizability of our findings. Third, although immunohistochemistry (IHC) is a widely accepted method for protein expression assessment, it is inherently semi-quantitative and subject to inter-observer variability. Although we utilized standardized scoring protocols and blinded evaluation by multiple independent reviewers to minimize variability, some subjectivity remains unavoidable. Moreover, while our in vitro experiments support the functional involvement of *GPAT4* and *SLC7A11* in platinum resistance, the underlying molecular mechanisms remain to be fully elucidated. Importantly, in vivo validation was not included in the current study. Future work using xenograft or genetically engineered mouse models will be critical to explore the therapeutic potential of combined inhibition of *GPAT4* and *SLC7A11*. Finally, external validation in larger, independent patient cohorts and prospective clinical studies will be essential to confirm the prognostic and predictive value of *GPAT4/SLC7A11* co-expression, particularly in the context of platinum resistance in epithelial ovarian cancer (EOC). Although time-dependent ROC curves and C-index analyses may offer additional prognostic insights, the current study focused primarily on conventional survival analysis methods to assess the clinical relevance of *GPAT4* and *SLC7A11.* Future investigations may build on our findings by incorporating dynamic prediction models and time-to-event ROC analyses in larger, prospectively collected datasets. Continuously, our follow-up study will provide insights into the mechanisms by which *GPAT4* regulates platinum resistance in ovarian cancer, adding prospective evaluation, increasing the number of patients with EOC, and establishing a link between *SLC7A11* and the regulation of *GPAT4*.

This study has demonstrated that the expression of *GPAT4* is an independent predictive factor for EOC survival. Low expression of *GPAT4* contributes to immediate sensitivity to platinum-based chemotherapy in ovarian cancer. Moreover, the combination of *GPAT4* and *SLC7A11* was found to be a novel marker predicting platinum resistance in ovarian cancer. This study indicates that the co-expression pattern of *GPAT4* and *SLC7A11* is a better indicator of resistance than the expression of either marker alone. This study highlights the potential value of *GPAT4* or *SLC7A11* as therapeutic targets for EOC; specifically, the results indicate that targeting *GPAT4* or *SLC7A11* may increase the effectiveness of platinum-based chemotherapy. Identifying patients with high *GPAT4/SLC7A11* expression may lead to tailored treatment options and improve patient outcomes. Future studies should be performed to verify the clinical importance of *GPAT4* and *SLC7A11* in predicting platinum resistance in EOC, and the development of targeted therapies or combination therapies to modulate their activity is underway.

## 5. Conclusions

High expression of both *GPAT4* and *SLC7A11* serves as an independent prognostic factor for patients with platinum-resistant EOC. The findings of this study indicate the potential value of *GPAT4* and *SLC7A11* as therapeutic targets.

## Data Availability

The raw data supporting the conclusions of this article will be made available by the authors on request.

## Supplementary Materials

The following supporting information can be downloaded at https://www.mdpi.com/article/10.3390/biomedicines13071664/s1, Table S1: Clinical characteristics of 50 high-grade serous ovarian cancer patients undergoing 5hmc sequencing; Table S2: Association of GPAT4 expression with clinicopathological parameters; Table S3: Association of SLC7A11 expression with clinicopathological parameters; Table S4: ROC analysis of the association of *GPAT4* and *SLC7A11* with platinum resistance; Table S5: Logistic regression analysis of the association of *GPAT4* and *SLC7A11* with platinum resistance. Figure S1 OS comparison of *GPAT4* and *SLC7A11* between the high-score group and low-score group of TCGA.
