# Supplementary material for: High Co-Expression of GPAT4 and SLC7A11 as a Predictor of Platinum Resistance and Poor Prognosis in Patients with Epithelial Ovarian Cancer
Source: Biomedicines. 2025 Jul 8;13(7):1664. doi: 10.3390/biomedicines13071664 (PMC12292186; doi:10.3390/biomedicines13071664)
Supplement: Supplementary file 1 [file biomedicines-13-01664-s001.zip › biomedicines-3692077-supplementary.pdf]

biomedicines

MDPI

Table S1. Clinical characteristics of 50 high-grade serous ovarian cancer patients undergoing 5hmc sequencing.

**Variable**	**Platinum sensitive, n (%)**	**Platinum resistant, n (%)**	***p*-value**
Number of patients age, years	35(70.00)	15(30.00)	
<50	13(37.14)	3(20.00)	0.234
≥50	22(62.86)	12(80.00)	
FIGO stage			
III	33(94.29)	12(80.00)	0.123
IV	2(5.71)	3(20.00)	
Serum CA125			
<100 U/ml	5(14.29)	1(6.67)	0.447
≥100 U/ml	30(85.71)	14(93.33)	
Ascitic fluid volume			
<500 ml	20(57.14)	6(40.00)	0.266
≥500 ml	15(42.86)	9(60.00)	
Number of chemotherapy required for CA125 to restore normal			
<3	26(74.29)	4(26.67)	0.002**
≥3	9(25.71)	11(73.33)	
R0-R1/R2			
R0-R1	32(91.43)	9(60.00)	0.008**
R2	3(8.51)	6(40.00)	

*$p < 0.05$; **$p < 0.01$; ***$p < 0.001$

Table S2. Association of GPAT4 expression with clinicopathological parameters.

Variable	GPAT4-low expression, n (%)	GPAT4-high expression, n (%)	*p*-value
Number of patients age, years	69(67.65)	33(32.35)	
<50	19(27.53)	8(24.24)	0.910
≥50	50(72.46)	25(75.80)	
FIGO stage			
I/II	8(11.59)	4(12.12)	1.000
III/IV	61(88.41)	29(87.88)	
Serum CA125			
<100 U/ml	10(14.49)	3(9.09)	0.654
≥100 U/ml	59(85.51)	30(90.91)	
Ascitic fluid volume			
<500 ml	39(56.50)	15(45.50)	0.403
≥500 ml	30(43.50)	18(54.50)	
Number of chemotherapies required for CA125 to restore normal			
＜3	39(56.52)	12(36.36)	0.090
≥3	30(43.48)	21(63.64)	
R0-R1/R2			
R0-R1	59(85.55)	20(60.61)	0.010*
R2	10(14.45)	13(39.39)	
Platinum resistance			
Sensitive	59 (85.51)	9(27.27)	＜0.001***
Resistant	10(14.49)	24(72.73)	

*$p < 0.05$; **$p < 0.01$; ***$p < 0.001$

Table S3. Association of SLC7A11 expression with clinicopathological parameters

Variable	SLC7A11-low expression, n (%)	SLC7A11-high expression, n (%)	*p*-value
Number of patients age, years	63(61.76)	39(38.24)	
<50	18(28.57)	9(23.08)	0.704
≥50	45(71.43)	30(76.92)	
FIGO stage			
I/II	8(12.70)	4(10.26)	0.956
III/IV	55(87.30)	35(89.74)	
Serum CA125			
<100 U/ml	7(11.11)	6(15.38)	0.746
≥100 U/ml	56(88.89)	33(84.62)	
Ascitic fluid volume			
<500 ml	32(50.79)	22(56.41)	0.728
≥500 ml	31(49.21)	17(43.59)	
Number of chemotherapies required for CA125 to restore normal			
<3	33(52.38)	18(46.15)	0.577
≥3	30(47.62)	21(53.85)	
R0-R1/R2			
R0-R1	51(80.95)	28(71.79)	0.406
R2	12(19.05)	11(28.21)	
Platinum resistance			
Sensitive	53(84.13)	24(61.54)	0.019*
Resistant	10(15.87)	15(38.46)	

*$p < 0.05$; **$p < 0.01$; ***$p < 0.001$

Table S4. ROC analysis of the association of GPAT4 and SLC7A11 with platinum resistance.

	AUC	*p*-value	95% CI
GPAT4 IHC score	0.917	<0.001***	0.865-0.977
SLC7A11 IHC score	0.908	<0.001***	0.815-1.000
Co GPAT4-SLC7A11	0.874	<0.001***	0.772-0.975
GPAT4 group	0.874	<0.001***	0.772-0.975
SLC7A11 group	0.874	<0.001***	0.772-0.975

*$p < 0.05$; **$p < 0.01$; ***$p < 0.001$

Table S5. Logistics regression analysis with platinum resistance.

Factors	Waldχ^2	OR	*P*-value
GPAT4	2.795	16.357	< 0.001**
SLC7A11	1.809	6.104	0.002**
R0/R1	-1.516	0.220	0.034*
FIGO stage	1.328	3.775	0.177

*$p < 0.05$; **$p < 0.01$; ***$p < 0.001$

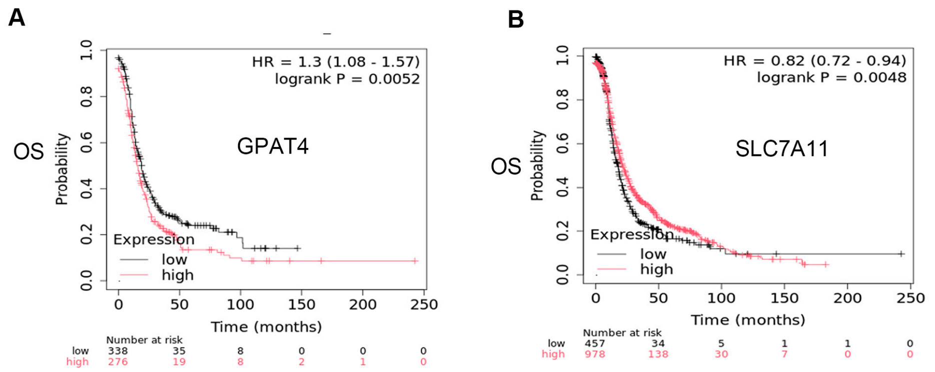

Figure S1 OS comparison of *GPAT4* and *SLC7A11* between high score group and low score group. (A) OS comparison between *GPAT4* high score group and *GPAT4* low score group (TCGA). (B) OS comparison between *SLC7A11* high score group and *SLC7A11* low score group (TCGA).
